# Longitudinal Trends in Blood Pressure Associated With the Frequency of Laughter: The Circulatory Risk in Communities Study (CIRCS), a Longitudinal Study of the Japanese General Population

**DOI:** 10.2188/jea.JE20190140

**Published:** 2021-02-05

**Authors:** Satomi Ikeda, Ai Ikeda, Kazumasa Yamagishi, Miyuki Hori, Sachimi Kubo, Mizuki Sata, Chika Okada, Mitsumasa Umesawa, Tomoko Sankai, Akihiko Kitamura, Masahiko Kiyama, Tetsuya Ohira, Takeshi Tanigawa, Hiroyasu Iso

**Affiliations:** 1 Department of Public Health, Juntendo University Graduate School of Medicine, Tokyo, Japan; 2 Department of Public Health Medicine, Faculty of Medicine, and Health Services Research Center, University of Tsukuba, Tsukuba, Japan; 3 Public Health, Department of Social Medicine, Osaka University Graduate School of Medicine, Osaka, Japan; 4 Department of Public Health, Dokkyo Medical University, School of Medicine, Tochigi, Japan; 5 Osaka Center for Cancer and Cardiovascular Diseases Prevention, Osaka, Japan; 6 Department of Epidemiology, Fukushima Medical University, Fukushima, Japan; 7 Research Team for Social Participation and Community Health, Tokyo Metropolitan Geriatric Hospital, Tokyo, Japan; 8 Cohort Study Team, Center for Cluster Development and Coordination, Foundation for Biomedical Research and Innovation at Kobe, Hyogo, Japan; 9 Department of Preventive Medicine and Public Health, Keio University School of Medicine, Tokyo, Japan

**Keywords:** laughter frequency, stress, cardiovascular-related biomarker, blood pressure, longitudinal study

## Abstract

**Background:**

The frequency of laughter has been associated with cardiovascular disease and related biomarkers, but no previous studies have examined association between laughter and changes in blood pressure levels. We sought to identify temporal relationships between frequency of laughter in daily life and systolic and diastolic blood pressure changes in participants from 2010 through 2014.

**Methods:**

Participants were 554 men and 887 women aged 40–74 years who answered self-administered questionnaire quantifying frequency of laughter at baseline. We measured participant blood pressure levels twice using automated sphygmomanometers for each year from 2010 to 2014. The associations between laughter and changes in blood pressure over time were analyzed using linear mixed-effect models.

**Results:**

There was no significant difference in blood pressure according to frequency of laughter at baseline in either sex. Men with frequency of laughter 1 to 3 per month or almost never had significantly increased systolic and diastolic blood pressure levels over the 4-year period (time-dependent difference: 0.96 mm Hg (95% confidence interval [CI], −0.2 to 1.8; *P* = 0.05). Changes in blood pressure associated with infrequent laughter (ie, 1 to 3 per month or almost never) were evident in men without antihypertensive medication use over 4 years (0.94 mm Hg; 95% CI, −0.2 to 2.0; *P* = 0.09) and men who were current drinkers at baseline (1.29 mm Hg; 95% CI, −0.1 to 2.3; *P* = 0.04). No significant difference was found between frequency of laughter and systolic (0.23 mm Hg; 95% CI, −1.0 to 1.5; *P* = 0.72) and diastolic (−0.07 mm Hg; 95% CI, −0.8 to 0.7; *P* = 0.86) blood pressure changes in women.

**Conclusions:**

Infrequent laughter was associated with long-term blood pressure increment among middle-aged men.

## INTRODUCTION

The proverb “laughter is the best medicine” has an element of truth, as laughter has been shown to relieve stress and positively impact mental stability. In 1976, Norman Cousins, a sufferer of ankylosing spondylitis, made the joyous discovery that 10 minutes of genuine belly laughter allowed him to sleep without pain for at least 2 hours. He subsequently adopted laughter as a treatment for his disease.^1^ The results of his blood test showed an improved erythrocyte sedimentation rate, suggesting that laughter not only removed pain but also had a positive impact on biomarkers.^1^ These findings have stimulated researchers to conduct intervention studies, which have shown that laughter improved symptoms of depression,^2^ insomnia,^3^ and natural killer cell activity,^4^^–^^6^ as well as reduction in HbA_1c_.^7^ A laughter and exercise program (ie, laughter yoga) has been demonstrated to increase bone mineral density and decrease HbA1c levels.^7^ An increased frequency of laughter may be useful for health promotion and motivate the elderly to enhance physical activity.

The frequency of laughter in everyday life is associated inversely with mental stress.^8^ Reduced mental stress may contribute to reduced activation of the hypothalamic-pituitary-adrenal axis and the sympathetic nervous system,^9^ as well as improve vascular endothelial function^10^ and heart rate variability.^11^ All of these effects may in turn reduce blood pressure levels.^12^ A previous study showed that the mean value of salivary cortisol concentration, a surrogate marker of mental stress, declined after the participants watched a comedy, and this value was particularly lower in both men and women who laughed everyday compared with those who laughed less often.^8^

Although various mechanisms have accounted for the association between laughter and cardiovascular disease and related biomarkers,^7^^,^^13^^,^^14^ no previous studies have examined the association between the frequency of laughter and blood pressure levels, an important risk factor for cardiovascular disease. If the beneficial impact of laughter on blood pressure levels is confirmed, psychological interventions to increase the frequency of laughter may contribute to the prevention of cardiovascular disease.

Therefore, in this longitudinal study, we examined the effect of laughter on blood pressure levels in the general population. We sought to examine our *a prior* hypothesis that lower frequency of laughter may lead to incremental increases in blood pressure levels. We aimed to identify the temporal relationship between the frequency of laughter in daily life and blood pressure levels over 4 years. Since lifestyles (ie, occupation and drinking and smoking status) and social psychological factors are largely different between men and women,^15^ we carried out sex-specific analyses.

## METHODS

### Study sample

The participants were the residents of Kyowa, a rural district of Chikusei City, Ibaraki Prefecture, Japan. The Kyowa community is included in the community-based part of the Circulatory Risk in the Community Study (CIRCS).^16^^–^^18^ Annual cardiovascular risk surveys have been conducted between mid-November and mid-December since 1981.^16^^,^^17^ The study subjects were residents aged 40–74 years who participated in the annual health checkups from 2010 through 2014 in the CIRCS.

In 2010, a baseline self-administered questionnaire quantifying the frequency of laughter was administered to 1,710 Kyowa residents (705 men and 1,005 women) aged 40–79 years old. We excluded 269 participants who had a history of cardiovascular disease (stroke and coronary artery disease) or were missing information on the frequency of laughter, age, or sex at the baseline. As a result, 1,441 participants (554 men and 887 women) remained eligible for the study from the baseline.

Furthermore, we separately analyzed participants with and without antihypertensive medication use between 2010 and 2014 and conducted the stratified analysis according to the baseline drinking status. Antihypertensive medication use was defined as “yes” in any of annual surveys between 2010 to 2014.

### Questionnaire survey on the frequency of laughter

In the questionnaire, we asked participants, “How often do you laugh out loud in your daily life?” to measure their frequency of laughter. Respondents could choose one of four possible answers: “almost every day”, “1 to 5 days per week”, “1 to 3 days per month”, or “almost never”. In this study, the categories of “1 to 3 days per month” and “almost never” were combined because only 36 participants (25 men and 11 women) answered “almost never”. We chose “almost every day” as the reference category.

The 1-year test–retest reliability of the questionnaire was assessed in a previous study of 2,680 men and women aged 30–74 years (the Spearman correlation coefficient = 0.61, *P* < 0.001).^19^ In addition, no regional or seasonal differences were found in the frequency of laughter among Japanese men and women.^20^ Therefore, the questionnaire on laughter is reliable and generalizable.

### Blood pressure measurement

All participants had their blood pressure levels measured twice using automated sphygmomanometers (TM-2655P; A&D Company Ltd. Tokyo, Japan) on their right arms once a year from 2010 to 2014.^17^ According to the validation study of the TM-2655 series, the device was awarded “A” grades by the British Hypertension Society for systolic and diastolic blood pressure measurements, and the proportion of values within 5 mm Hg was 72.5% for systolic blood pressure and 78.8% for diastolic blood pressure.^21^ We used averages of the first and second blood pressure measurements for each year from 2010 to 2014 for the analyses. In our study, a systolic blood pressure level of 140 mm Hg or higher and a diastolic blood pressure level of 90 mm Hg or higher were defined as hypertension.

From the baseline, 1,441 participants (554 men and 887 women) remained eligible for this study. The numbers of participants for subsequent annual follow-ups were 713 (49.5%) for all four follow-ups, 225 (15.6%) for three follow-ups, 157 (10.9%) for 2 follow-ups, 148 (10.3%) for 1 follow-up, and 198 (13.7%) for no follow-up. We included these 198 participants who were lost to follow-up in the analyses.

### Other covariates

We calculated participant body mass index (BMI) by dividing weight (kg) by the square of height (m^2^). Socks and light clothing were included when measuring height and weight. Waist measurements were made at the umbilical level and clothes were removed at the time of measurement. Each participant was interviewed to determine their usual weekly alcohol consumption in “go” units, a traditional Japanese unit of volume equivalent to 23 g of ethanol. Smoking status and history were also determined in the interview, as well as the present daily number of cigarettes smoked. Histories of hypertension, stroke, coronary heart disease, and use of antihypertensive medication were also collected during the interview.

Low-density lipoprotein (LDL) cholesterol (mg/dL), high-density lipoprotein (HDL) cholesterol (mg/dL), blood glucose levels (mg/dL), and factors related to cardiovascular disease were measured at the annual health examination. Fasting glucose ≥5.55 mmol/L (100 mg/dL), non-fasting glucose ≥7.77 mmol/L (140 mg/dL), HbA1c level ≥6.5%, and/or diabetes treatment were defined as hyperglycemia.

To measure participants’ mental health, we asked, “Do you feel stressed in your work or daily life?” and offered the following response options: “all of the time”, “much of the time”, “a little”, or “almost never”. We also asked, “Have you had little interest or pleasure in doing things in the past month?” and “Have you felt depressed or hopeless in the past month?”, to which participants responded with “yes” or “no”.

### Ethical approval

This study was approved by the ethics committees of the University of Tsukuba (12–6, 66–2), Osaka University (13482, 14285), and Ethics Review Board of Juntendo University’s Faculty of Medicine (2016091), and the institutional review boards of the Osaka Center for Cancer and Cardiovascular Disease Prevention (26-rinri-1). In accordance with Japanese ethical guidelines, we provided participants with information about our study at the survey sites and informed them of opt-out opportunities. All procedures performed in the studies involving human participants were in accordance with the ethical standards of the institutional and/or national research committee and with the 1964 Helsinki declaration and its later amendments or comparable ethical standards.

### Informed consent

We obtained informed consent for the use of existing data from representatives in communities, but not from each research participant since the current study is the secondary use of the data obtained for public health practice on cardiovascular disease prevention in local communities. However, participants were given the option to withdraw their consent. If the participant(s) did not clearly decline consent, their consent was granted in the present study.

### Statistical analysis

Age-adjusted means and proportions of various lifestyle and psychological factors were tested using analysis of covariance and by conducting chi-square tests of baseline characteristics. To examine the effect of changes in the frequency of laughter on changes in blood pressure over time, we analyzed the data using mixed effects modeling, the commonly accepted method for dealing with longitudinal data, which accounts for correlations among measurements taken from the same individual. In the present study, we used the following model (SAS MIXED procedure)^22^:

$$\begin{array}{rl} Y_{ij} & =(\beta_{0}+b_{\text{0i}})+\beta_{p}\cdot {\text{laughter}}_{i}+\beta_{T}\cdot {\text{Time}}_{ij}\\ & \;+\beta_{PT}(\text{laughter}\times \text{time}{)}_{ij}+\varepsilon_{ij}. \end{array}$$

where *y_ij_* represents the systolic and diastolic blood pressure levels for individual *i* taken at time *j*; β_0_ is the overall intercept; β*_p_* is the effect of the frequency of laughter, considered as constant across time; β*_T_* represent respectively the intercept and the slope of the linear relationship between the systolic and diastolic blood pressure levels and time at which the outcome was measured; and β*_PT_* is the effect of laughter frequency on the slope describing the linear relationship between blood pressure levels and time (blood pressure changes per year). Coefficients for this model were estimated by maximum likelihood using the SAS MIXED procedure and specifying a compound symmetry structure for the covariance matrix.^22^^–^^25^

The covariates included baseline age, time (the difference in age between the baseline and the time at which the outcome was measured), presence of stress at the baseline (all of the time, much of the time, a little, or almost never), presence of declining interest and depressed mood before the baseline (yes or no), participants’ baseline occupational status (managerial, professional, manual labor, sales and services, self-employed, farming, housewife, or unemployed), smoking status from 2010 to 2014 (yes or no), drinking status from 2010 to 2014 (never, ex-drinkers, current drinkers of ethanol at 1 to 22 g/day, or current drinkers of ethanol at ≥23 g/day), antihypertensive medication use from 2010 to 2014 (presence or absence of medication), and BMI (kg/m^2^) from 2010 to 2014. Covariate measurements were assessed at study baseline. When the frequency of laughter was missing, we used available measurements closest in time when the frequency of laughter was assessed. To assess the potential modifying effects of baseline age and change in age on the relation of the frequency of laughter with blood pressure levels over time, we ran regression models that included cross-product terms for interaction between age and change in age with the frequency of laughter along with the main-effects.

Furthermore, we separately analyzed participants with and without antihypertensive medication use between 2010 and 2014. Participants who had never used antihypertensive medication during the 4 years from the baseline were included in the group without antihypertensive medication use. Those who used antihypertensive medication from the baseline or during any follow-up surveys were included in the group with antihypertensive medication use. Because alcohol intake is a strong covariate for blood pressure levels,^26^ we conducted the stratified analysis according to the baseline drinking status.

We used SAS software version 9.4 (SAS Institute, Cary, NC, USA) for all analyses. A *P* value of <0.05 (two-tailed) was considered statistically significant, except for a *P* value <0.10 in the interaction analysis according to previous study.^25^^,^^27^

## RESULTS

Table 1 shows the sex-specific baseline characteristics for the frequency of laughter in everyday life. Men and women who answered that they did not laugh much tended to report declining interest and feelings of subjective stress and depression over the past month. Men who laughed less often were more likely to be current smokers.

**Table 1.** Sex-specific baseline characteristics by the frequency of laughter in everyday life (*n* = 1,441)

	Men (*n* = 554)				Women (*n* = 887)			

**Frequency of laughter**	Almost everyday	1 to 5 days per week	1 to 3 days a month or almost never	*P* for difference	Almost everyday	1 to 5 days per week	1 to 3 days a month or almost never	*P* for difference
**Number**	318	164	72		653	190	44	

**Age, mean (SD)**	63.3(10.9)	62.9(10.3)	63.5(9.5)	0.63	61.8(10.1)	61.0(9.4)	60.4(9.4)	0.44

**Waist, mean (SD)**	73.7(30.7)	75.1(30.6)	72.8(30.4)	0.78	73.8(26.6)	74.9(26.3)	77.1(23.4)	0.60

**BMI, kg/m^2^, mean (SD)**	23.6(2.9)	24.1(3.0)	23.2(2.6)	0.03	22.9(3.1)	22.8(3.2)	23.0(4.0)	0.98

**Systolic blood pressure, mean (SE)**	133.3(0.9)	133.8(1.4)	130.4(2.4)	0.31	124.6(0.7)	124.0(1.1)	123.0(2.4)	0.78

**Diastolic blood pressure, mean (SE)**	78.0(0.6)	78.9(0.9)	76.3(1.6)	0.23	73.2(0.5)	73.6(0.9)	71.6(1.4)	0.57

**Antihypertensive medication use, %**	30.1	27.1	33.7	0.56	25.8	24.4	25.0	0.97

**Hypertension, %**	50.9	49.4	48.6	0.90	37.8	37.4	34.1	0.87

**Hyperglycemia, %**	33.0	37.2	36.1	0.64	19.6	14.7	20.5	0.29

**Current smoker, %**	23.9	22.6	27.8	0.04	4.3	1.6	4.6	0.20

**Current drinker, %**	63.5	66.4	70.8	0.47	15.0	16.3	9.1	0.48

**Low HDL cholesterol, %**	6.9	7.3	4.2	0.65	11.3	17.8	15.5	0.05

**Occupation, %**								
**managerial**	1.0	0.0	0.0	0.33	0.0	0.0	0.0	
**professional**	0.3	0.6	1.4	0.52	1.9	0.0	0.0	0.09
**manual labor**	12.7	15.5	14.3	0.77	5.5	6.4	13.6	0.13
**sales and services**	3.8	2.5	1.4	0.48	8.8	7.4	4.6	0.39
**self-employed**	18.4	17.4	17.1	0.91	11.2	5.8	6.8	0.05
**farming**	29.4	27.3	22.9	0.53	18.6	20.6	9.3	0.21
**housewife**	0.6	0.0	2.9	0.06	39.2	47.1	54.5	0.01
**unemployed**	33.9	36.7	40.0	0.43	14.8	12.7	11.4	0.69

**Subjective mental stress, %**	12.6	12.2	22.2	0.01	12.4	22.6	36.3	<0.001

**Depressed in mood, %**	3.4	5.4	14.0	0.01	3.2	8.5	22.7	<0.001

**Declining interest, %**	3.5	5.5	12.5	0.01	4.3	5.3	13.6	0.03

BMI, body mass index; HDL, high-density lipoprotein; SD, standard deviation; SE, standard error.

Hypertension: systolic pressure ≥140 mm Hg or diastolic pressure ≥90 mm Hg, and/or antihypertensive medication use, Hyperglycemia: fasting glucose ≥5.55 mmol/L (100 mg/dL), or non-fasting glucose ≥7.77 mmol/L (140 mg/dL) and/or on treatment or non-fasting glucose ≥7.77 mmol/L (140 mg/dL) and/or on treatment or HbA1c ≥6.5%, Low HDL cholesterol: HDL cholesterol <1.03 mmol/L (40 mg/dL) for men and <1.29 mmol/L (50 mg/dL) for women.

*P* for difference: adjusted for age.

The *P*-value for the difference is based on the assessment comparing between three groups by using the analysis of covariance.

Table 2 shows the sex-specific changes in systolic and diastolic blood pressure over time according to the frequency of laughter in everyday life. At the baseline, there was no significant difference in systolic and diastolic blood pressures according to the frequency of laughter at the baseline in either sex. Men who answered that their frequency of laughter was 1 to 3 days per month or almost never had increased systolic blood pressure (time-dependent difference: β = 0.96 mm Hg; 95% CI, −0.2 to 1.8; *P* = 0.05) and diastolic blood pressure (time-dependent difference: β = 0.72 mm Hg; 95% CI, 0.1–1.2; *P* = 0.02) compared with men with the frequency of laughter ≥1 day/week. In this group, mean systolic blood pressure at the baseline in 2010 was 130.7 mm Hg and increased to 134.1 mm Hg in 2014. Mean diastolic blood pressure was 75.9 mm Hg at the baseline and increased to 78.9 mm Hg in 2014. In women, the mean systolic and diastolic blood pressure did not change over the 4 years in any group.

**Table 2.** Sex-specific changes in systolic and diastolic blood pressures with time by the frequency of laughter in everyday life

	Men					Women				

	Frequency of laughter					Frequency of laughter				

	Almost everyday (reference)	1 to 5 days per week		1 to 3 days a month or almost never		Almost everyday (reference)	1 to 5 days per week		1 to 3 days a month or almost never	
Number	318	164		72		653	190		44	

		β (95% CI)	*P* value	β (95% CI)	*P* value		β (95% CI)	*P* value	β (95% CI)	*P* value

**Systolic blood pressure**										
Baseline difference	0	0.75(−1.9, 3.9)	0.62	−2.38(−6.5, 1.7)	0.26	0	1.10(−1.4, 3.6)	0.39	0.64(−4.2, 5.4)	0.79
Time-dependent difference^**^	0	−0.34(−1.1, 0.3)	0.34	0.96(−0.2, 1.8)	0.05^*^	0	−0.11(−0.7, 0.5)	0.74	0.23(−1.0, 1.5)	0.72
Mean value for 2010, (SD)	133.2(1.0)	133.1(1.3)		130.7(2.0)		124.4(0.7)	124.9(1.2)		124.4(2.5)	
Mean value for 2014, (SD)	133.0(1.1)	131.4(1.4)		134.1(2.2)		125.0(0.7)	124.9(1.2)		126.9(3.0)	

**Diastolic blood pressure**										
Baseline difference	0	−0.16(−1.9, 2.0)	0.87	−2.09(−4.8, 0.7)	0.14	0	0.86(−0.9, 2.6)	0.34	−0.06(−4.0, 2.7)	0.72
Time-dependent difference^**^	0	−0.07(−0.5, 0.4)	0.74	0.72(0.1, 1.2)	0.02^*^	0	−0.31(−0.7, 0.1)	0.11	−0.07(−0.8, 0.7)	0.86
Mean value for 2010, (SD)	77.0(0.6)	77.4(0.8)		75.9(1.2)		72.8(0.4)	73.4(0.8)		72.1(1.5)	
Mean value for 2014, (SD)	76.6(0.6)	76.8(0.8)		78.9(1.3)		73.7(0.5)	72.7(0.8)		71.8(1.9)	

CI, confidence interval.

^*^P value of interaction with time.

^**^Time × the frequency of laughter.

Adjusted for age, baseline occupational status (managerial, professional, manual labor, sales and services, self-employed, farming, housewife or unemployed), antihypertensive medication use from 2010 to 2014, presence of mental stress at baseline, depressed in mood and declining interest at baseline, current smoking, drinking status and BMI from 2010 to 2014.

The *P*-value of interaction with time is based on the assessment comparing between reference category (“almost every day”) and other categories by using the linear mixed-effect models.

Table 3 shows the sex-specific changes in systolic and diastolic blood pressure over time according to the frequency of laughter in everyday life with and without antihypertensive medication use. In men without antihypertensive medication use who had the frequency of laughter of ≥1 day/week, systolic or diastolic blood pressure did not change over 4 years. However, men who had the frequency of laughter of 1 to 3 days per month or almost never had significant increases over time in both systolic blood pressure (time-dependent difference: β = 0.94 mm Hg; 95% CI, −0.2 to 2.0; *P* = 0.09) and diastolic blood pressure (time-dependent difference: β = 0.82 mm Hg; 95% CI, 0.1–1.5; *P* = 0.02) over time. In this group, mean systolic blood pressure at the baseline in 2010 was 129.8 mm Hg and increased to 134.3 mm Hg in 2014. We found a similar association for diastolic blood pressure (75.4 mm Hg at the baseline and 78.6 mm Hg in 2014). However, no significant difference in blood pressure change was evident according to the frequency of laughter in men with antihypertensive medication use and in women with or without antihypertensive medication use.

**Table 3.** Sex-specific changes in systolic and diastolic blood pressures with time by the frequency of laughter in everyday life with and without antihypertensive medication use

	Men					Women				

	Frequency of laughter					Frequency of laughter				

	Almost everyday (reference)	1 to 5 days per week		1 to 3 days a month or almost never		Almost everyday (reference)	1 to 5 days per week		1 to 3 days a month or almost never	
	No antihypertensive medication users									

**Number**	237	124		49		498	147		32	

		β (95% CI)	*P* value	β (95% CI)	*P* value		β (95% CI)	*P* value	β (95% CI)	*P* value

**Systolic blood pressure**										
Baseline difference	0	0.79(−2.4, 4.5)	0.66	−2.58(−7.8, 2.5)	0.33	0	1.20(−1.7, 4.1)	0.42	−2.01(−7.8, 3.8)	0.50
Time-dependent difference^**^	0	−0.39(−1.2, 0.4)	0.33	0.94(−0.2, 2.0)	0.09^*^	0	0.13(−0.5, 0.8)	0.70	0.07(−1.3, 1.4)	0.92
Mean value for 2010, (SD)	131.9(1.1)	132.1(1.5)		129.8(2.5)		122.5(0.8)	123.5(1.4)		120.4(3.0)	
Mean value for 2014, (SD)	133.2(1.3)	131.0(1.7)		134.3(2.8)		123.7(0.9)	125.1(1.6)		124.2(3.6)	

**Diastolic blood pressure**										
Baseline difference	0	0.32(−2.1, 2.7)	0.98	−2.81(−6.4, 0.6)	0.12	0	0.72(−1.3, 2.7)	0.48	−0.96(−4.9, 3.0)	0.64
Time-dependent difference^**^	0	0.10(−0.4, 0.6)	0.69	0.82(0.1, 1.5)	0.02^*^	0	−0.17(−0.6, 0.3)	0.43	−0.02(−0.9, 0.8)	0.97
Mean value for 2010, (SD)	77.6(0.7)	77.9(1.0)		75.4(1.7)		71.9(0.5)	72.7(0.9)		70.8(2.0)	
Mean value for 2014, (SD)	77.6(0.7)	78.0(1.1)		78.6(1.9)		72.9(0.6)	72.4(0.9)		72.5(2.4)	

	Antihypertensive medication users									

**Number**	81	40		23		155	43		12	

		β (95% CI)	*P* value	β (95% CI)	*P* value		β (95% CI)	*P* value	β (95% CI)	*P* value

**Systolic blood pressure**										
Baseline difference	0	1.99(−3.2, 7.6)	0.47	−0.49(−7.6, 6.0)	0.89	0	−1.10(−5.6, 3.4)	0.63	3.65(−4.5, 11.6)	0.37
Time-dependent difference^**^	0	0.09(−1.4, 1.7)	0.90	1.08(−1.2, 2.7)	0.29	0	−0.48(−1.9, 1.1)	0.54	−0.46(−3.2, 2.4)	0.75
Mean value for 2010, (SD)	139.0(1.8)	140.0(2.4)		136.6(3.3)		132.9(1.2)	130.5(2.3)		134.2(4.4)	
Mean value for 2014, (SD)	134.9(1.9)	136.8(2.4)		135.2(3.7)		132.8(1.3)	128.4(2.5)		131.3(5.5)	

**Diastolic blood pressure**										
Baseline difference	0	0.96(−2.2, 4.4)	0.57	0.04(−4.2, 4.0)	0.99	0	−0.09(−3.3, 3.1)	0.95	−1.61(−7.4, 4.1)	0.59
Time-dependent difference^**^	0	−0.21(−1.0, 0.7)	0.62	0.54(−0.7, 1.4)	0.34	0	−0.42(−1.3, 0.5)	0.36	−0.37(−2.0, 1.3)	0.66
Mean value for 2010, (SD)	77.8(1.0)	79.5(1.4)		78.7(1.9)		76.6(0.8)	76.2(1.5)		73.6(3.0)	
Mean value for 2014, (SD)	76.8(1.1)	77.2(1.4)		79.5(2.2)		78.2(0.9)	75.7(1.7)		73.5(3.7)	

CI, confidence interval; SD, standard deviation.

^*^P value of interaction with time.

^**^Time × the frequency of laughter.

Adjusted for age, baseline occupational status (managerial, professional, manual labor, sales and services, self-employed, farming, housewife or unemployed), antihypertensive medication use from 2010 to 2014, presence of mental stress at baseline, depressed in mood and declining interest at baseline, current smoking, drinking status and BMI from 2010 to 2014.

The *P*-value of interaction with time is based on the assessment comparing between reference category (“almost every day”) and other categories by using the linear mixed-effect models.

Sex-specific changes in systolic and diastolic blood pressure over time according to the frequency of laughter in everyday life by baseline drinking status are shown in eTable 1. The associations between the lower frequency of laughter and the increment of systolic and diastolic blood pressure levels were confined to current drinkers in men.

eTable 2 shows sex-specific changes in systolic and diastolic blood pressures with time by the frequency of laughter in everyday life without controlling for psychological conditions (eg, stress, declining interest and depressed mood). The associations between the lower frequency of laughter and the increment of systolic and diastolic blood pressure levels in men did not change materially.

## DISCUSSION

In this study, we found no significant difference in systolic and diastolic blood pressure levels according to the frequency of laughter at the baseline. However, men who replied that they laughed “1 to 3 days per month” or “almost never” had significantly increased systolic and diastolic blood pressure over the 4-year period compared to those who answered that they laughed “almost every day” or “1 to 5 days per week”. We found no significant change over time for either systolic or diastolic blood pressure in women. The effect of the frequency of laughter on blood pressure increment was more evident in men without antihypertensive medication use during the study period and men with current drinking status at the baseline.

A plausible mechanism for the association between the frequency of laughter and blood pressure may be through the effect of mental stress.^8^^,^^28^ The relationship between laughter and mental stress has been documented in previous intervention studies. A randomized controlled trial was conducted in Korean breast cancer patients using a four-session therapeutic laughter program (*n* = 31) to examine the effect of mental health status (ie, anxiety, depression, and stress levels) compared with control patients (*n* = 29).^29^ Each therapeutic laughter session was a 60-minute class of laughing in rhythm with clapping, laughing for a long time, laughing with the whole body, laughing in various ways, and laughing together with dance routines. In that trial, participants showed significantly lower anxiety, depression, and stress levels after the first laughter session, which remained thereafter.^29^ Another Iranian randomized controlled trial with the laughter therapy program (ie, two 90-minute sessions per week performing breathing and physical exercises and laughter for 6 weeks) found a significant increase in general health scores (*P* = 0.001) and a decrease in somatic symptoms scores (*P* = 0.001), as well as insomnia and anxiety scores (both *P* = 0.001).^30^

Another mechanism for the association between the frequency of laughter and blood pressure levels may be through the improvement of vascular endothelial function. A cross-over trial of 17 apparently healthy adults (23–42 years of age) investigated the relationship between laughter and vascular function.^31^ The trial participants watched a 30-minute comedy or documentary on separate days, which led to the brachial artery flow mediated dilation (FMD), a marker of endothelial function, increasing significantly after watching a comedy and reducing after watching a documentary.^31^ Reduced FMD was associated with higher systolic and diastolic blood pressure levels,^32^ which is a known predictor of future cardiovascular events.^33^

Previous studies found no association of the laughter frequency with the prevalence of hypertension at baseline.^14^^,^^34^ In our longitudinal study, however, the lower frequency of laughter at baseline was associated with the overtime increase in systolic and diastolic blood pressure among men. Our study is the first to find longitudinal association between infrequent laughter and blood pressure increases.

We found no significant association between the frequency of laughter and blood pressure changes in women. One of the explanations for the lack of association for women may be much lower means level of systolic and diastolic blood pressures at the baseline in women than in men, which made it difficult to detect any effect of laughter on blood pressure changes. Another explanation may be the effect modification by alcohol consumption on the association between laughter and blood pressure changes. The significant positive association between infrequent laughter and blood pressure increment was confined to current drinkers in men probably because alcohol consumption increases blood pressure levels which may likely detect that association or because of unknown reasons.^26^ Only 9% of women were current drinkers; thus, the association between infrequent laughter and blood pressure increment was unlikely to appear.

A strength of the present study is a prospective study with a large sample size to identify the association between the frequency of laughter and blood pressure levels over a period of 4 years. Most of the subjects (86.3%) had more than one blood pressure measurement between 2010 and 2014. Moreover, there was no significant difference in baseline characteristics, including the frequency of laughter and baseline blood pressure levels, between subjects who had more than one blood pressure measurement and those who only had the baseline blood pressure measurement (not shown in tables). Therefore, the withdrawal from the follow-up was unlikely to affect the results.

The present study has several limitations. First, the frequency of daily laughter was evaluated using a single self-reported question. It is possible that the perceived frequency of laughter differs from actual frequency. Second, we only had information regarding declined interest, feelings of subjective stress, and depression at the baseline. Therefore, we could not consider changes in the negative emotional status from 2011 to 2014 in the analyses. Furthermore, all the answers to questionnaire were self-reported and we used a single simple question rather than scores about negative emotions, such as self-perceived mental stress, depressed mood, and declined interest. We also lacked objective data on negative emotions. Third, although previous studies showed that higher income and high social participation were significantly associated with higher frequency of laughter,^19^^,^^35^ we did not collect the information on education status, income, or other socioeconomic factors other than occupational status as potential confounders, so we could not rule out the possibility of residual confounding. Fourth, we could not consider the effect of positive emotions other than laughter, such as contentment, amusement, and optimism due to the lack of data. Lastly, the effect of infrequency laughter on blood pressure increment was more evident in men without antihypertensive medication use during the study period. However, the respective samples with antihypertensive medication use for the frequency of laughter of “1 to 3 days per month” or “almost never” were unlikely to have enough statistical power to detect any significant effects. Statistical power with alpha 5% using a MIXED model was 68% for a time-dependent difference of systolic blood pressure and 57% for that of diastolic blood pressure.

In conclusion, the present longitudinal observational study suggests that infrequent laughter leads to the increment of blood pressure levels during a 4-year period among middle-aged men. Future studies should investigate whether psychological interventions are useful for preventing or controlling hypertension in men who laugh infrequently.

## APPENDIX A. SUPPLEMENTARY DATA

eTable 1. Sex-specific changes in systolic and diastolic blood pressures with time by the frequency of laughter in everyday life according to drinking status

eTable 2. Sex-specific changes in systolic and diastolic blood pressures with time by the frequency of laughter in everyday life without controlling for psychological conditions
