# Supplementary material for: Longitudinal Trends in Blood Pressure Associated With the Frequency of Laughter: The Circulatory Risk in Communities Study (CIRCS), a Longitudinal Study of the Japanese General Population
Source: J Epidemiol. 2021 Feb 5;31(2):125–31. doi: 10.2188/jea.JE20190140 (PMC7813767; doi:10.2188/jea.JE20190140)
Supplement: Supplementary file 1 [file je-31-125-s001.pdf]

eTable 1. Sex-specific changes in systolic and diastolic blood pressures with time by the frequency of laughter in everyday life according to drinking status

	Men					Women				
	Frequency of laughter					Frequency of laughter				
	Almost everyday (reference)	1 to 5 days per week		1 to 3 days a month or almost never		Almost everyday (reference)	1 to 5 days per week		1 to 3 days a month or almost never	
Non current drinkers										
**Number**	116	55		21		555	159		40	
		β (95% CI)	p value	β (95% CI)	p value		β (95% CI)	p value	β (95% CI)	p value
**Systolic blood pressure**										
Baseline difference	0	-0.59(-5.1, 4.0)	0.80	-3.97(-10.3, 3.0)	0.28	0	1.02(-1.6, 3.7)	0.45	0.03(-4.9, 5.0)	0.99
Time-dependent difference**	0	0.09(-1.1, 1.2)	0.89	0.04(-1.7, 1.9)	0.97	0	-0.24(-0.9, 0.5)	0.49	0.07(-1.2, 1.4)	0.92
**Diastolic blood pressure**										
Baseline difference	0	1.27(-1.6, 4.5)	0.42	-4.05(-9.0, 0.7)	0.11	0	0.79(-1.1, 2.7)	0.41	-0.19(-3.7, 3.3)	0.91
Time-dependent difference**	0	-0.18(0.9, 0.5)	0.62	0.05(-1.1, 1.2)	0.93	0	-0.37(-0.8, 0.1)	0.09	-0.12(-09, 0.7)	0.77
Current drinkers										
**Number**	202	109		51		98	31		4	
		β (95% CI)	p value	β (95% CI)	p value		β (95% CI)	p value	β (95% CI)	p value
**Systolic blood pressure**										
Baseline difference	0	0.41(-3.1, 4.4)	0.83	-2.04(-7.1, 3.0)	0.43	0	1.17(-5.2, 7.4)	0.71	6.59(-8.5, 21.7)	0.39
Time-dependent difference**	0	-0.49(-1.5, 0.4)	0.29	1.29(-0.1, 2.3)	0.04*	0	0.31(-1.3, 2.0)	0.71	0.78(-4.8, 6.4)	0.78
**Diastolic blood pressure**										
Baseline difference	0	-1.35(-3.7, 1.3)	0.29	-2.10(-5.3, 1.4)	0.22	0	1.45(-3.3, 5.9)	0.54	-1.49(-12.5,9.5)	0.79
Time-dependent difference**	0	-0.03(-0.6, 0.5)	0.93	0.96(0.1, 1.6)	0.01*	0	-0.29(-1.3, 0.8)	0.59	0.76(-2.9, 4.5)	0.69

CI, confidence interval; SD, standard deviation.

* p value of interaction with time

** Time×the frequency of laughter

eTable 2. Sex-specific changes in systolic and diastolic blood pressures with time by the frequency of laughter in everyday life without controlling for psychological conditions

	Men					Women				
	Frequency of laughter					Frequency of laughter				
	Almost everyday (reference)	1 to 5 days per week		1 to 3 days a month or almost never		Almost everyday (reference)	1 to 5 days per week		1 to 3 days a month or almost never	
Number	318	164		72		653	190		44	
		β (95% CI)	*p* value	β (95% CI)	*p* value		β (95% CI)	*p* value	β (95% CI)	*p* value
**Systolic blood pressure**										
Baseline difference	0	0.72(-2.2, 3.7)	0.62	-3.6(-7.6, 0.4)	0.17	0	0.64(-1.8, 3.1)	0.61	-0.36(-5.1, 4.3)	0.88
Time-dependent difference**	0	-0.39(-1.1, 0.3)	0.34	0.83(-0.1, 1.8)	0.08*	0	-0.14(-0.8, 0.5)	0.65	0.24(-1.0, 1.5)	0.71
**Diastolic blood pressure**										
Baseline difference	0	-0.08(-0.5, 0.3)	0.73	-2.67(-5.3, 0.1)	0.06	0	0.67(-1.1, 2.4)	0.45	-0.99(-4.3, 2.3)	0.56
Time-dependent difference**	0	-0.07(-0.5, 0.4)	0.74	0.65(0.1, 1.2)	0.03*	0	-0.34(-0.7, 0.4)	0.08	-0.08(-0.8, 0.7)	0.85

CI, confidence interval.

* *p* value of interaction with time

** Time×the frequency of laughter
